# Evidence of a Protein-Coding Gene Antisense to the U_L_5 Gene in Bovine Herpesvirus I

**DOI:** 10.3390/v15101977

**Published:** 2023-09-22

**Authors:** Victoria A. Jefferson, Hannah Bostick, Darby Oldenburg, Florencia Meyer

**Affiliations:** 1 Department of Biochemistry, Molecular Biology, Entomology & Plant Pathology, Mississippi State University, 32 Creelman St., Starkville, MS 39762, USA; vaj13@msstate.edu (V.A.J.); hannah.bostick.04@gmail.com (H.B.); 2 Gundersen Medical Foundation, 1900 South Ave., La Crosse, WI 54601, USA; dgoldenb@gundersenhealth.org

**Keywords:** bovine herpesvirus, novel protein, transcript, homolog

## Abstract

Bovine herpesvirus type 1 (BoHV-1) is an important agricultural pathogen that infects cattle and other ruminants worldwide. Though it was first sequenced and annotated over twenty years ago, the Cooper strain, used in this study, was sequenced as recently as 2012 and is currently said to encode 72 unique proteins. However, tandem mass spectrometry has identified several peptides produced during active infection that align with the BoHV-1 genome in unannotated regions. One of these abundant peptides, “ORF M”, aligned antisense to the DNA helicase/primase protein U_L_5. This study characterizes the novel transcript and its protein product and provides evidence to support the existence of homolog protein-coding genes in other Herpesviruses.

## 1. Introduction

Bovine herpesvirus type 1 (BoHV-1) is a critical viral pathogen that affects ruminants all over the world [1,2,3,4]. It is one of several viruses that contribute to the Bovine Respiratory Disease complex (BRD), which costs the cattle industry billions through prevention, treatment, and production losses [5,6,7,8]. Acute BoHV-1 infection, together with a physiological response to stress, typically leads to secondary bacterial infections from commensal organisms such as *Mannheimia haemolytica*, *Pasteurella multocida*, or *Histophilus somni* which can cause pneumonia and increased risk of mortality [9]. Around 28% of dairy calf morbidity and 14% of dairy calf mortality cases can be attributed solely to respiratory illness [10]. As a herpesvirus, BoHV-1 presents a unique risk through its ability to establish latency in the trigeminal ganglia and tonsils [11,12]. Frequent stress to the host can cause reactivation of the virus throughout life, leading to a renewed risk of BRD.

Bovine herpesvirus 1 (BoHV-1) belongs to the subfamily *Alphaherpesvirinae* within the Varicellovirus genus [4,13]. It, like other herpesviruses, is composed of a double-stranded DNA genome enclosed within an icosahedral nucleocapsid, surrounded by an amorphous tegument layer, and enveloped by a host cell-derived double lipid bilayer. BoHV-1 has a genome of around 135 kb and is known to encode 72 unique proteins. It was initially sequenced and annotated via an international effort using strains from both 1.1 and 1.2a subtypes [3,14,15,16,17]. The Cooper strain of subtype 1.1 was sequenced individually in 2012, but the process of updating and expanding the current annotations is ongoing [18]. Genome-wide multiple sequence alignment analysis of recent BoHV-1.1 isolates illustrates differences such as insertions, deletions, and point mutations between both the original composite sequence and each other using resources available from the Bacterial and Viral Bioinformatics Resource Center [19,20].

Increased availability of advanced sequencing and analysis technologies has allowed an influx of discovery within herpesvirus genomes. In other alphaherpesviruses, such as pseudorabies virus (PRV), the discovery of novel coding and noncoding transcripts, transcript isoforms as well as complex polygenic transcripts produced during infection have led to improved accuracy of the structure of the PRV transcriptome [21,22]. Two recently identified noncoding RNAs produced by PRV, although not essential for virulence, appear to be involved in transcriptional regulation and may affect viral DNA replication [23]. In the case of Herpes Simplex Virus type 1 (HSV-1), which has a similar genome architecture as BoHV-1, post-translational modifications such as ubiquitylation and phosphorylation were identified using proteomic analysis [24]. Evidence of these modifications provided an expanded list of targets to further unravel HSV-1 regulatory mechanisms [25,26]. In human cytomegalovirus (HCMV), evidence of novel RNAs has been verified using techniques such as RACE, while deep RNA sequencing identified novel splice sites and many ncRNAs antisense to previously described coding regions [27]. In the Epstein–Barr virus (EBV), these same technologies have revealed novel splice isoforms and alternative transcription start and stop sites [28]. Collectively, these studies have added nuance to the architecture of herpesviruses’ genomes and highlight the complexity and coding potential contained in their sequences.

The study of the BoHV-1 transcriptional landscape has also benefited from advances in sequencing technology in the last decade. Evidence of alternative transcriptional start and stop sites, as well as previously unknown complexities in its transcription kinetics, has recently been described in multiple studies [29,30,31]. Examples of these discoveries are the alternative start and stop codons in genes bICP22, U_L_10, and U_L_26.5–26 and many transcripts with extended 5′ ends [30]. Our 2018 study revealed the existence of several mass-spectrometry peptides that mapped to unannotated regions of the genome [32]. We hypothesize that BoHV-1 partakes in the production and translation of several antisense transcripts and proteins. In this study, we characterize the transcript as well as the protein product of a gene encoding an ORF antisense to the U_L_5 gene.

## 2. Materials and Methods

### 2.1. Cells and Virus

Madin-Darby Bovine Kidney (MDBK) cells were grown using Dulbecco’s modified Eagle’s medium (DMEM) supplemented with 100 U/mL penicillin and 100 mg/mL streptomycin and 5% (*v*/*v*) fetal bovine serum (FBS). Bovine turbinate (BT) cells were grown with Dulbecco’s modified Eagle’s medium (DMEM) supplemented with 100 U/mL penicillin and 100 mg/mL streptomycin but with 10% (*v*/*v*) FBS. Cells were maintained in a 37 °C humidified incubator with 5% CO_2_ levels. Bovine Herpesvirus 1.1 Cooper isolate (GenBank Accession number JX898220.1) was used for infections.

### 2.2. Infection

Cells were washed in phosphate-buffered saline (PBS) and incubated in serum-free DMEM with antibiotics and BoHV-1 at 4 °C for one hour, shaking every 15 min. Cells were washed with PBS again before adding fresh DMEM and returning to the 37 °C incubator. MDBK and BT cells were mock-infected or infected at a multiplicity of infection (MOI) of 5 for 4, 8, 12, 16, 20, and 24 h. After infection, plates were washed with PBS, scraped, and centrifuged for 5 min at 8000× *g* and stored at −20 °C for mRNA or protein extraction.

### 2.3. RNA Extraction

Messenger RNA (mRNA) was isolated from mock and infected BT and MDBK using a magnetic mRNA isolation kit (#S1550S, New England Biolabs, Ipswich MA, USA). To remove genomic DNA contamination, mRNA was treated with DNase I following the manufacturer’s instructions (#EN0521, Thermo Fisher Scientific, Waltham, MA, USA).

### 2.4. 5′ RACE, 3′ Primer Walking, Regular and Quantitative (q)RT-PCR

Rapid Amplification of cDNA Ends (RACE) was performed using the Invitrogen GeneRacer Kit (#L1502-01, Invitrogen, Thermo Fisher Scientific). After ligation of the provided RNA-oligo to the 5′ ends of mRNA, synthesis of cDNA was carried out using gene-specific primers synthesized by Eurofins (Louisville, KY, USA) to amplify the 5′ end of the ORF M transcript (Table 1). mRNA extracted from MDBK cells infected at an MOI of 5 for 24 h was used as the template for cDNA synthesis. Primers specific to the RNA-oligo (provided by the manufacturer) and user-designed gene-specific primers were used to amplify the 5′ sequence through polymerase chain reaction (PCR).

**Figure 1:**
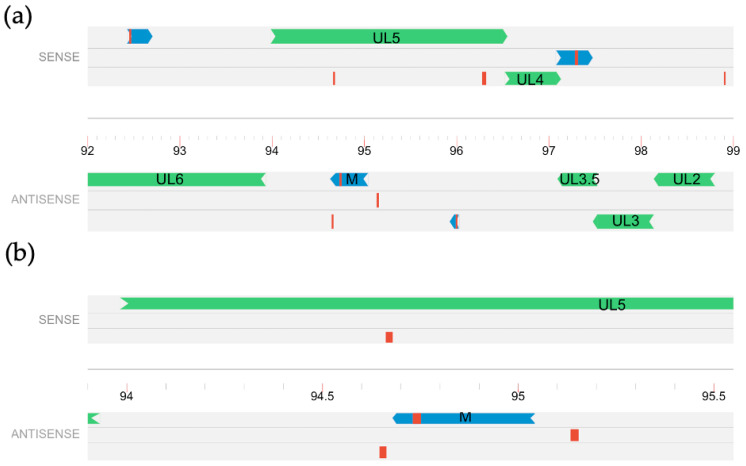
Relative location of predicted ORF M. Six reading frames of a portion of the BoHV-1.1 genome, represented as a single line at the center, with tick marks showing genomic coordinates in kilo-bases. (**a**) Peptides identified via MS/MS are shown in red and predicted open reading frames around them are shown in dark blue. Green represents known annotated ORFs; (**b**) A magnified portion of the BoHV-1.1 genome that contains ORF M with nearby MS/MS peptides.

Multiple primers were designed increasingly further downstream to map the 3′ end of the transcript (Figure 1b; Table 1). mRNA from MDBK cells infected at an MOI of 5 for 24 h was used as a template to generate strand-specific cDNA with each reverse primer in a separate reaction. The Invitrogen Superscript III module (#18080-44, Carlsbad, CA, USA) was used as recommended. Each cDNA was used as an individual template to amplify the 3′ end with a common forward primer. Negative controls were conducted by using the DNase-treated mRNA as a template instead of the cDNA to discard the possibility of genomic DNA contamination.

PCR products were visualized using 1% agarose gels in TAE buffer (40 mM Tris (pH 7.6), 20 mM acetic acid, and 1 mM EDTA). Bands for the 5′ RACE and 3′ primer walking amplicons were excised with a clean scalpel and purified using the Monarch nucleic acid purification kit (#T1020, New England Biolabs). Purified DNA was quantified using a NanoDrop spectrophotometer. The 5′ and 3′ amplicons were sequenced and compared to the full BoHV-1 Cooper sequence using NCBI BLASTn (Accession: JX898220.1).

For transcription kinetics, the Invitrogen Superscript III module was used to generate cDNA from mock and BoHV-1 MDBK cells collected at 4, 8, 12, 16, and 24 h post-infection (HPI). Gene/strand-specific primers (Eurofins) were used to generate ORF M cDNA, while random hexamers (Invitrogen) were used for cDNA synthesis of cellular and viral genes. All reverse transcriptase PCR (RT-PCR) was carried out using the OneTaq module (#M0480S, New England Biolabs). Negative controls used a template mRNA untreated with reverse transcriptase.

For quantitative (q) PCR, cDNA templates were used with primers designed to create smaller amplicons (Eurofins) that allow for more efficient DNA extension (Table 1). Samples were generated from mock and MDBK cells infected with BoHV-1.1 for 2, 4, 6, and 8 h. The HOT FIREPol EvaGreen qPCR mix with ROX was used for all qPCR applications (#08-24-00001, Solis BioDyne, Tartu, Estonia). Negative controls used mock and 8 HPI samples that went untreated with reverse transcriptase as a template. Results were analyzed using the QuantStudio Design and Analysis Software v2.6.0 (Applied Biosystems, Thermo Fisher Scientific).

### 2.5. Immunoblot

Infected MDBK and BT cell pellets were incubated on ice for 30 min in lysis buffer (50 mM Tris-HCl, 150 mM NaCl, 0.5% NP-40, cOmplete mini protease inhibitor cocktail [#04693159001, Basel, Switzerland]). Pellets were vortexed and spun down for 5 min at 8000× *g*. Protein concentrations were quantified from supernatants using Bradford assay (#5000002, BioRad, Hercules, CA, USA). Twenty micrograms of protein per sample were heated to 85 °C for 5 min in 2X Tris-glycine SDS buffer (#LC2676, Novex, Invitrogen) and separated using SDS polyacrylamide gel electrophoresis (PAGE) at 100 V for 90 min. Proteins were transferred from the gel to a polyvinylidene fluoride membrane (#IPVH00010, MilliporeSigma, Burlington, MA, USA) via wet electroblotting at 200 mAmps for 120 min, and the membrane was blocked for 1 h at room temperature in blocking buffer (5% non-fat dry milk (NFDM) in Tris-HCl buffered Saline (TBS) (50 mM Tris-HCl pH 7.4, 150 mM NaCl) supplemented with 0.05% Tween-20 (TBS-T)).

The ORF M MS/MS peptide sequence TGMAPSR was the basis for synthesizing an antigenic polypeptide, which was used to inoculate rabbits (Boster Biological Technology, Pleasanton, CA, USA). Two New Zealand white rabbits were immunized with subcutaneous peptide antigen (0.5 mg) and boosted on days 21, 35 and 50 with 0.25 mg of antigen. Antiserum was affinity purified with antigen and tested by ELISA. The rabbit polyclonal antibody was diluted 1:500 and used to probe the immunoblots at 4 °C overnight. Blots were washed with TBS-T three times for fifteen minutes each and then probed with anti-rabbit secondary antibody diluted to 1:2000 (#sc-2357, Santa Cruz Biotechnologies, Dallas, TX, USA) in blocking buffer for one hour at room temperature. The blot was washed again 3 times with TBS-T for fifteen minutes each. Blots were visualized using the Pierce ECL Western Blotting Substrate (#32106, Thermo Fisher Scientific).

### 2.6. Immunocytochemistry

Cells were seeded on 4 well glass slides (Millipore) and infected at an MOI of 1 for 20 h. At various times post-infection, slides were gently washed with PBS and fixed with ice-cold methanol for 20 min at −20 °C. After washing again with PBS, they were blocked at room temperature for 1 h with 3% bovine serum albumin (BSA) diluted in PBS. Slides were then probed with the ORF M antibody at a concentration of 1:100 in 1% BSA in TBS-T overnight at 4 °C. After washing gently with TBS-T 3 times for 15 min each, the slides were incubated at room temperature for 1 h in the dark with Alexa Fluor 488 conjugated anti-Rabbit antibody (#A-11034, Invitrogen, Thermo Fisher Scientific) diluted to 1:100 in 1% BSA in TBS-T. After washing again with PBS 3 times for 15 min each, the slides were mounted using a coverslip and DAPI gel mounting medium (#F6057, Sigma-Aldrich, Darmstadt, Germany) and visualized using a Zeiss Axio Scope.A1 microscope and DAPI and FITC filters.

### 2.7. Bioinformatic Analysis

Using the Minimap2 aligner [33], BoHV-1.1 transcripts extracted from MDBK cells infected at an MOI of 5 between 1 to 12 h (Accession: PRJEB33511) were aligned to the BoHV-1.1 genome (Accession: JX898220.1) [33]. Transcript features were annotated using the LoRTIA toolkit developed by Zsolt Balázs of the Boldogkői laboratory at the University of Szeged, Hungary. The Samtools suite was used to convert files into bam format, index sequences, and select antisense reads in the genomic region that overlapped the estimated range of the ORF M sequence: 94,212–95,279 [34,35]. This range was chosen to ensure the capture of reads that map to our target region without the overrepresentation of irrelevant reads. Filtered out were also reads that spanned a region larger than 10 kb of the reference, as these largely consist of small regions that align to the reference, separated by large gaps. Filtered bam files were parsed in R using rtracklayer and visualized using Gviz [36,37]. Read coverage for this region at 2, 4, 6, 8, and 12 HPI were visualized separately in R using ggbio [38].

Using the default parameters in the NCBI BLAST suite, the full ORF M predicted nucleotide sequence was used for nucleotide queries. However, for protein queries, the translated RNA sequence from the predicted start to the predicted stop codons was used. MAFFT v7.490 [39] was used with the standard parameters to create a multisequence alignment with similar peptides identified via BLAST. The nucleotide and protein alignments were visualized using the ESPript 3.0 software [40].

## 3. Results

Our previous study used tandem mass spectrometry to identify peptides produced by BoHV-1 during productive infection of MDBK cells [32]. These peptide sequences were aligned to the BoHV-1 genome through proteogenomic mapping, a process that involves in silico translation of the BoHV-1 Cooper genome into all six reading frames, creating a proteogenomic database of all potential viral translations [41]. Experimental MS/MS peptides were then mapped onto the in silico database. The analysis identified at least 92 unique peptides that were mapped outside of currently annotated ORFs. For 21 of the unique peptides, a potential open reading frame was identified by scanning for in-frame start and stop codons using the genome visualizer Artemis v18.2.0 [42,43,44,45]. We focused on one abundant peptide, which we have arbitrarily named “ORF M”, located antisense to a major vial gene U_L_5 (Figure 1). In a previous study, we used strand-specific cDNA synthesis and PCR to verify the existence of transcripts produced by this region as well as other predicted ORFs [32]. We focused on this ORF as a potential protein-coding ORF for further characterization.

### 3.1. Evidence of Antisense Transcripts in the U_L_5 Region

**Figure 2:**
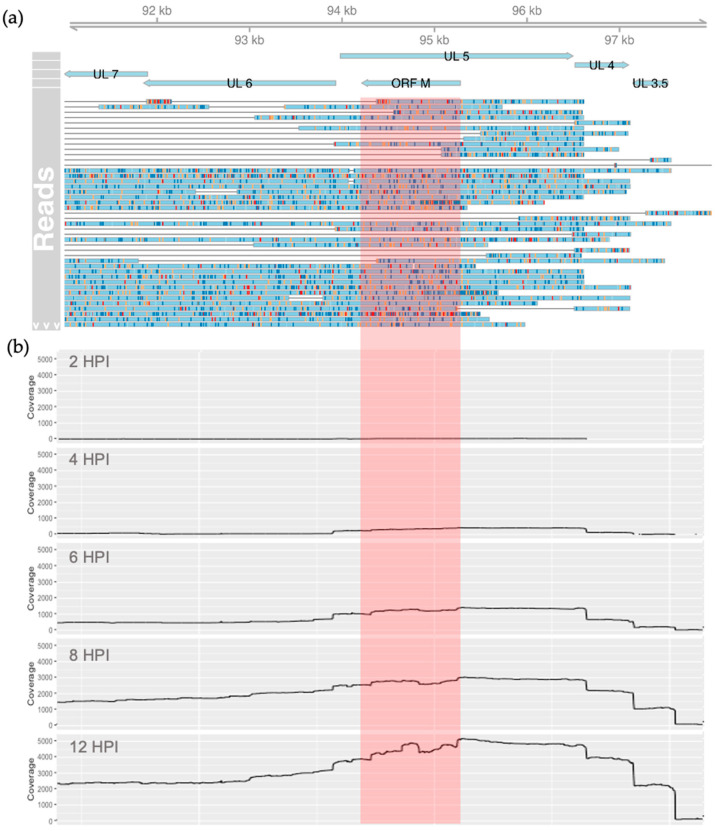
Transcripts detected in the genomic region between 94,212 and 95,279 of the BoHV-1.1 genome. (**a**) ORF M’s relevant coordinates are highlighted in red. Reads mapping to the antisense strand produced throughout infection are shown in light blue with mismatches colored red, gold, navy, and green for adenine, cytosine, thymine, and guanine, respectively. (**b**) Coverage of antisense reads overlapping the region between 94,212 and 95,279 at 2, 4, 6, 8, and 12 HPI. The region we associate with ORF M is highlighted in red.

To substantiate the existence of transcripts antisense to U_L_5, we used sequencing data from a recent collaborative project, where we conducted a whole transcriptomic analysis of BoHV-1.1 infected MDBK cells [29,30]. These data consisted of transcripts produced at 0, 1, 2, 4, 6, 8, and 12 h after BoHV-1.1 infection at an MOI of 5. Using these data, we searched for reads overlapping the genomic coordinates 94,212 to 95,279 (the predicted coordinates of the ORF M transcript), mapping antisense to U_L_5. We confirmed the existence of RNA transcripts, which can potentially drive the synthesis of the ORF M protein. A total of 1261 reads overlapped with the predicted ORF M coordinates (Figure 2a), 642 of which were detected at 12 h. Interestingly, a large amount of these reads were longer and extended downstream into the currently annotated gene U_L_6. In addition, a few transcripts also extended upstream into the U_L_3.5 gene. Both U_L_6 and U_L_3.5 are encoded in the same (antisense) strand (Figure 1 and Figure 2a). The coverage data generated from these reads illustrated an increase in transcription rates from this region as infection progressed (Figure 2b).

### 3.2. Characterization of the ORF M Transcript

**Figure 3:**
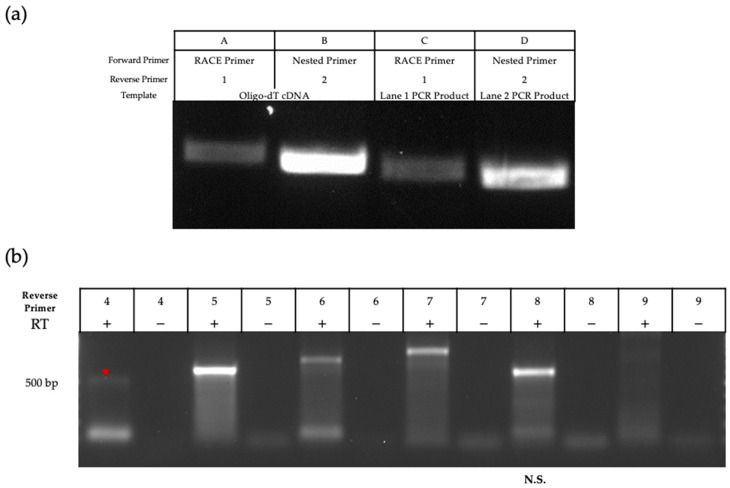
Amplification of ORF M cDNA ends. (**a**) 5′ RACE was executed using a strand-specific 3′ primer (lanes A and C). See Table 1 for primers. These amplicons were used as templates with nested primers (lanes B and D); (**b**) Primers designed increasingly further downstream of the 5′ RACE product were used to amplify the 3′ end of the ORF M transcript. The red star indicates the detection of a faint 500 bp amplicon. Amplification was specific to the region of interest until primer 7. Negative controls used mRNA that did not undergo cDNA synthesis as a template.

To elucidate the complete sequence of this transcript, we used rapid amplification of cDNA ends (RACE) to map/elucidate the 5′ end of the transcript (Figure 3a). cDNA synthesis was completed for mRNA extracted from MDBK cells infected at an MOI of 5 for 24 h. The RACE approach uses a proprietary primer that can bind to the 5′ Cap structure (RACE Primer, Figure 1b). To carry out RT-PCR, we designed 3′ primers in/around the region where the MS/MS peptide was mapped (Figure 1b). Successful amplification was achieved with two of the 3′ primers (Figure 3a). Nested primers were also designed to further amplify the initial RT-PCR products in cases of poor yield, and these primers also produced successful (and smaller) amplicons. After verification with agarose gel electrophoresis, amplicons were excised, purified, and sent for sequencing. BLASTn analysis further supported that the amplicon mapped to ORF M on the BoHV-1 genome, with transcription beginning around position 94,212 on the reverse strand.

**Figure 4:**
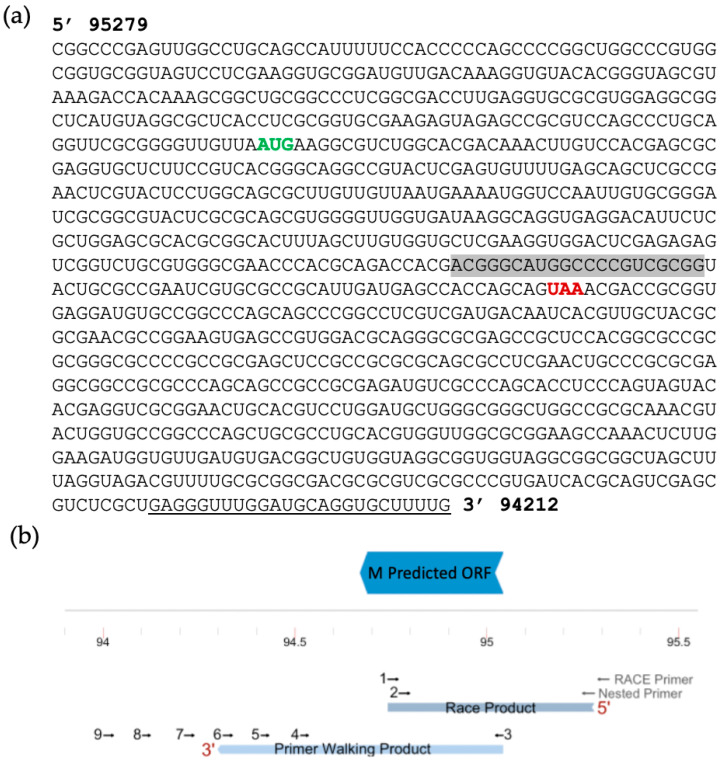
Predicted Full ORF M transcript. (**a**) Nucleotide sequence after sequencing and alignment of the RACE and primer walking PCR products to the BoHV-1.1 genome. The predicted in-frame start and stop codons around the ORF M MS/MS peptide are shown in green and red, respectively. The shaded sequence correlates to the location of the original peptide found via MS/MS. (**b**) Diagram of the predicted ORF with primers used for RACE and primer walking at their approximate target region. The mapping of the PCR products from both procedures is shown in light blue.

We attempted amplification of the 3′ end of the transcript via RACE but failed to produce an amplicon. Instead, we used primer walking as described previously [32]. Reverse primers were designed in a stepwise manner to cover the downstream sequence. Starting with infected cell-mRNA (MOI 5, 24HPI), we used location- and strand-specific primers to produce cDNA to ensure that only transcripts generated from the antisense strand were reverse transcribed and not any U_L_5 transcripts. This method was successful in amplification through reverse primer 7 (Figure 3b and Figure 4b for primer location). The amplicon produced using reverse primer 8 was smaller than expected when considering primer coordinates, and no clear band was amplified with reverse primer 9. All amplicons were excised and sequenced. Using NCBI BLASTn, all retrieved sequences were mapped to BoHV-1 except for the amplicon produced by primer 8, confirming that this sequence was non-specific. Therefore, we concluded that the amplicon produced using primer 7 represents the region where the transcript likely ends. Using both RACE and primer walking, the estimated length of the ORF M transcript is 1067 bp, located from nucleotide 94,212 to 95,279 (Figure 4). This estimation was possible due to the large overlap in the sequences of the 3′ and 5′ transcript analysis.

**Figure 5:**
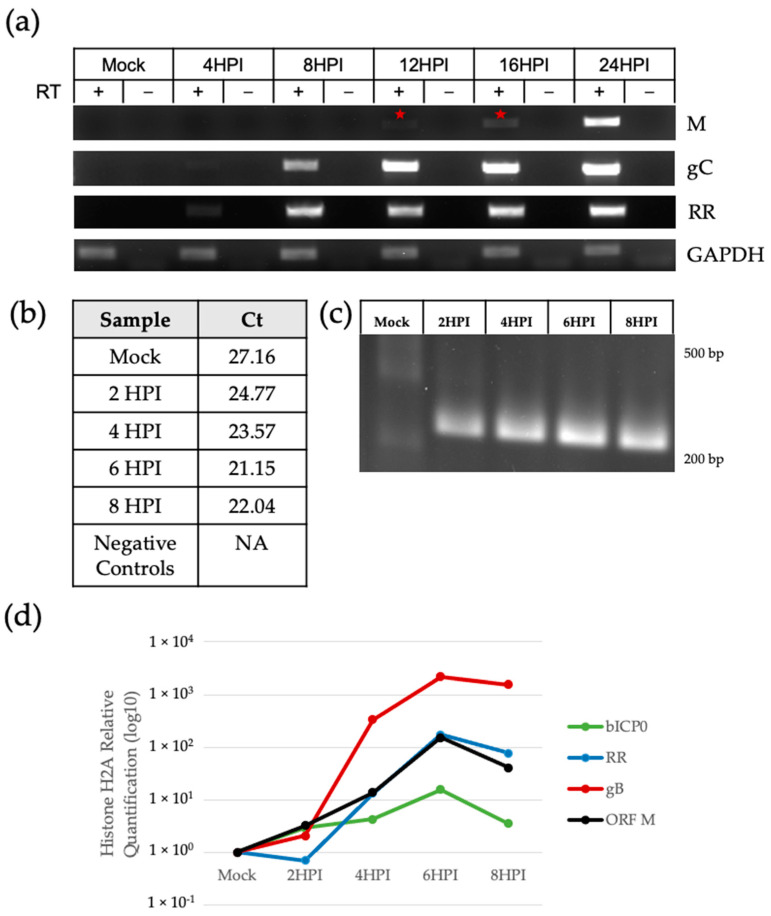
ORF M transcript kinetics. (**a**) Amplification of the ORF M transcript using strand-specific RT-PCR. RT-PCR for viral genes RR and gC and host GAPDH used random hexamers. Negative controls are PCR reactions using RNA that did not undergo cDNA synthesis (RT-). The red star indicates the earliest observation of an amplicon. (**b**) qPCR Ct values were recorded for mock and infected samples. Negative controls included RT-mock and 8 HPI samples; (**c**) qPCR products in (**b**) visualized in agarose gel. (**d**) Relative quantification of ORF M, bICP0, RR, and gB from (**c**) was achieved using the QuantStudio Design and Analysis Software 2.6.0.

Next, we determined the kinetics of ORF M transcription. Herpesvirus genes can be transcribed at immediate-early, early, or late times post-infection [46]. Immediate-early genes consist of transactivators such as bICP0 or bICP4 that activate early viral gene expression and viral replication. Late genes encode structural components, such as nucleocapsid proteins and envelope glycoproteins, which together make up the viral particle. Amplification of the ORF M transcript was seen as early as 12 HPI using regular RT-PCR, indicated by a red star in Figure 5a. Ribonucleotide reductase (RR) and glycoprotein C (gC) were used as early and late gene controls, respectively.

When using qRT-PCR, ORF M transcripts were detected at earlier time points post-infection (Figure 5b–d). Low levels of the transcript were detected as early as 2 HPI, with levels increasing over time. Accordingly, Figure 5b shows an inverse correlation between HPI and Ct values. DNA from mock-infected samples had non-specific amplification (Ct of 27.16), indicated by the lack of a clear amplicon (Figure 5c), in addition to melting curves with multiple irregular small peaks (Supplementary Figure S1). Using histone H2A as a host housekeeping gene, Figure 5d displays the relative quantification of the ORF M transcript levels compared to immediate early gene bICP0, early gene RR, and late gene gB.

### 3.3. Detection of the ORF M Polypeptide

**Figure 6:**
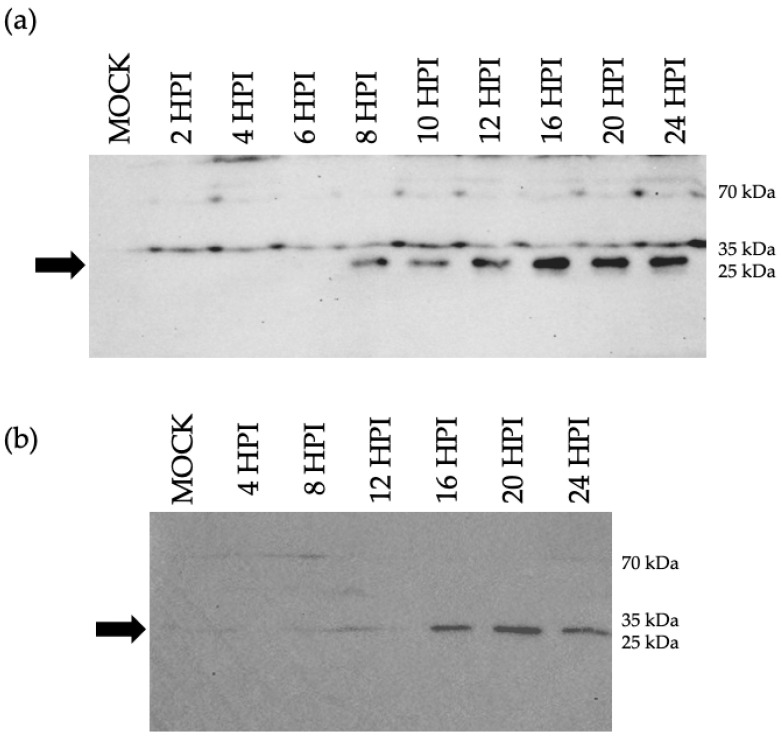
Immunoblot for detection of ORF M protein. Cells were infected with BoHV-1 at MOI 5 in a synchronized way for the indicated time (4–24 HPI). Uninfected cells (mock) served as a control. A total of 20 µg of whole cell lysates per lane were separated via SDS-PAGE. Blots were probed with an ORF M-specific antibody. The arrow points at a protein of about 30 kDa identified as early as 8 HPI in MDBK (**a**) and 16 HPI in BT (**b**).

An underlying assumption of this work is that the peptide identified via MS/MS was likely a fragment of a larger protein encoded by the putative ORF M gene. The MS/MS peptide was the basis for the design and synthesis of an antigenic polypeptide, which was used to generate a rabbit polyclonal antibody. Proteins from BoHV-1.1 and mock-infected MDBK cell lysates were separated by size through SDS-PAGE. Using the custom anti-ORF M antibody as a probe, a protein of around 30 kDa was detected beginning at 8 HPI (Figure 6a). When using a bovine respiratory cell line for the infection (BT cells), a 30 kDa protein was detected by 16 HPI (Figure 6b). The use of BT cells is relevant due to their respiratory origin and, therefore, provides a more relevant cellular environment for BoHV-1.1 replication than MDBK cells.

**Figure 7:**
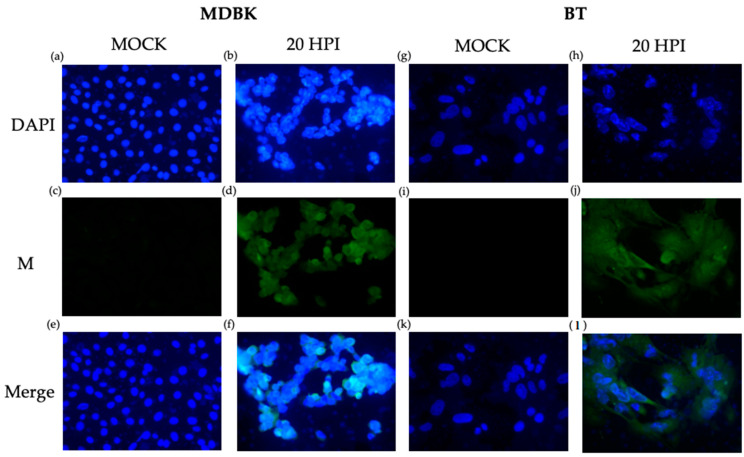
Detection of ORF M protein using immunocytochemistry. ORF M (green) was detected at 20 HPI using a custom anti-ORF M antibody and secondary conjugated to Alexa Fluor 488 in both MDBK (**a**–**f**) and BT cells (**g**–**l**). Nuclei were probed with DAPI, shown in blue. Total magnification 400×.

The same anti-ORF M antibody was also used for immunocytochemistry for further validation. Both MDBK and BT cells were infected at an MOI of 1. A fluorescent signal was detected in both infected MDBK and BT cells at 20 HPI (Figure 7). Earlier time points provided no clear signals. ORF M protein appeared to be distributed evenly throughout the cytoplasm and nucleus. In each multi-chamber slide, mock-infected cells located directly next to infected cells did not emit green fluorescence, indicating the absence of the ORF M protein. Mock-infected cells also did not show signs of virus-induced cytopathic effects (CPE), such as cell clumping and distorted nuclei. However, the CPE was apparent in infected cells, as evidenced by distorted and shrunk nuclei seen with the blue DAPI stain. Overall, we conclude that ORF M protein is produced and detectable in infected cells by 20 HPI and is distributed throughout the cell.

### 3.4. Identification of Homologous Herpesvirus Sequences

**Figure 8:**
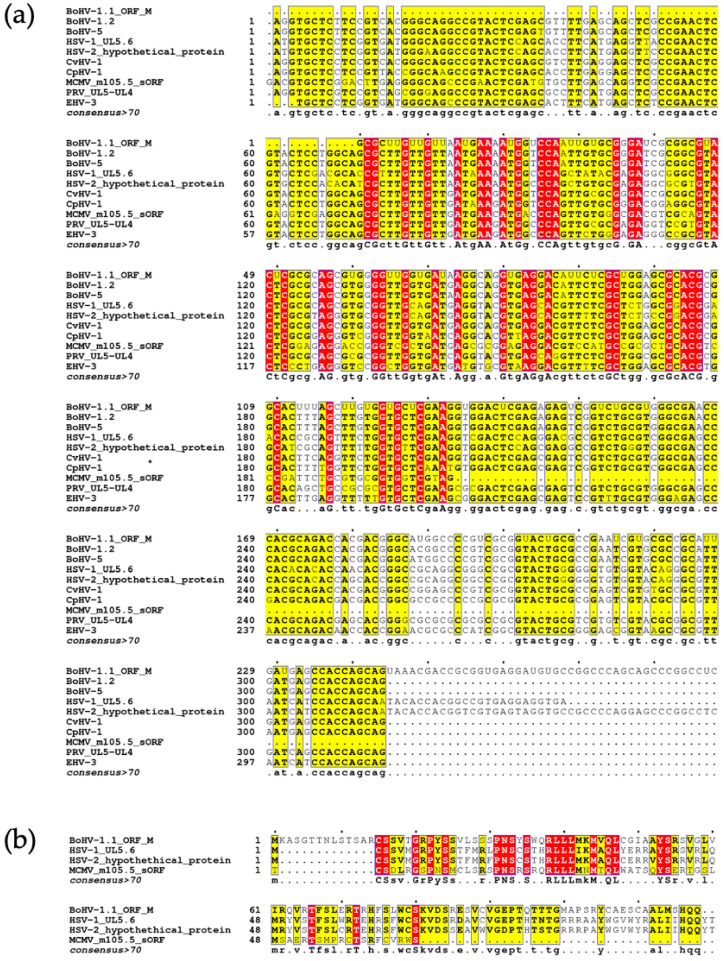
ORF M BLAST analysis. (**a**) Nucleotide sequence alignment of BoHV-1.1 ORF M, HSV-1, HSV-2, MCMV, BoHV-1.2, BoHV 5, CvHV 1, CpHV 1, PRV and EHV 3 using MAFFT. (**b**) Amino acid sequence alignment multiple sequence alignment between ORF M, HSV-1 UL 5.6, a hypothetical protein produced by HSV-2, and MCMV 105.5 sORF using MAFFT. In both alignments, the consensus sequence was generated using residues that were represented more than 70% between sequences.

Finally, we utilized the NCBI BLAST suite to identify sequences with similarity to ORF M. Because ORF M lies antisense to the U_L_5 gene, using the full ORF M nucleotide sequence as a query for BLASTn, BLASTx or tBLASTx overwhelmed the results with U_L_5 gene sequences encoded by the sense strand produced by the many BoHV-1.1 isolates in the database. We used tBLASTn to identify herpesviral sequences that could translate into similar proteins. A multisequence alignment was generated for the most similar sequences from individual herpesvirus subtypes (Figure 8a). These include nucleotide sequences from BoHV-1.2, BoHV-5, cervid herpesvirus 1, caprine herpesvirus 1, pseudorabies virus, and equine herpesvirus 3 (Accessions: OP035381.1, MW829288.1, NC_075564.1, NC_076509.1, L20708.1, and NC_076964.1 respectively). The ORF M amino acid sequence we have identified is much longer than any of the available sequences identified. For simplicity, the alignment shown consists of the trimmed portion of ORF M that is aligned with the other sequences. However, the full alignment can be found in the supplementary material (Figures S2 and S3). These nucleotide sequences all share at least 61% sequence identity with ORF M.

BLASTp uses the amino acid sequence from the translation of the ORF M nucleotide sequence for the search. Using this strategy, three similar proteins were identified: a hypothetical protein produced by HSV-2 uploaded from an unpublished study (Accessions: QBH76086.1), the HSV-1 U_L_5.6 protein identified through a large functional genomic study of HSV-1 (Accession: DAC85450.1) [47], and the murine cytomegalovirus (MCMV) m105.5 short ORF protein (Accession: DBA07604.1) [48]. To visualize the similarities of retrieved sequences, an amino acid multisequence alignment was generated using MAFFT (Figure 8b). The HSV-2 hypothetical protein shared around 58% sequence identity with ORF M, and HSV-1 U_L_5.6 shared over 60% sequence identity with ORF M. The MCMV 105.5 protein shares over 48% sequence identity. A common consensus for labeling proteins as homologous is having a sequence identity of greater than 30% [49]. The high nucleotide and amino acid identity shared with the three herpesviral proteins provides strong evidence for the existence of a protein-encoding gene in BoHV-1.

## 4. Discussion

In this study, we have provided evidence of a protein-coding gene in a previously unannotated region of the BoHV-1 genome. We refer to it as ORF M. This ORF is antisense to the essential gene U_L_5 that codes for a component of the helicase-primase complex [50]. The nucleotide sequence of this region is highly similar to other subtypes of BoHV-1 and closely related herpesviruses such as caprine (sheep and goat), cervid (deer), suid (boar), and equine alphaherpesviruses. Because U_L_5 is an essential gene, it is not surprising that this sequence is highly conserved. However, protein sequences encoded in the antisense strand may not necessarily maintain the same level of conservation (Supplementary Figure S5).

The finding that closely related alphaherpesviruses produce similar proteins identified through BLASTp [47,51] further supports the existence of ORF M. Whisnant and colleagues refer to the HSV-1 protein as U_L_5.6 (Accession: DAC85450.1), and Roychoudhury and colleagues refer to the HSV-2 sequences as “hypothetical proteins” (Accessions: QBH78348.1, QBH76086.1). The HSV-1 and HSV-2 proteins are found in similar structural regions in their respective genomes as with BoHV-1, and they, along with the MCMV m105.5 protein, are located antisense to helicase-primase complex-associated proteins. Because of synteny and high sequence identity, ORF M is a strong candidate to be a one-to-one ortholog gene in alphaherpesviruses. The similarity found with the MCMV protein further supports the conservation of these sequences since MCMV belongs taxonomically to the Betaherpesvirinae subfamily (as opposed to the Alphaherpesvirinae subfamily for BoHV-1, HSV-1, and HSV-2). The potential function of these proteins has not been determined yet, as these findings were parts of much larger, exploratory studies.

Our study adds to the ever-expanding hidden genetic potential within these relatively large DNA viral genomes. Herpesviruses have genomes of between 108 and 241 kb with hundreds of coding genes [52,53,54], but not all of them appear to be essential to in vitro and/or in vivo replication [55,56,57,58]. Due to the availability of increasingly advanced high-throughput sequencing and data analysis software, many herpesviruses have been shown to have increased transcriptional potential with both coding and noncoding transcripts. For example, Whisnant et al. identified 284 potential ORFs coded by HSV-1, much more than its currently annotated 80 genes [47]. Many of the newly discovered ORFs are antisense to larger genes such as U_L_5.6, ICP0, and ICP34.5. Whisnant’s study also confirms non-canonical events in cellular processes, such as alternative translation start codons, as well as alternative transcription and translation start sites. For example, 15–20% of coding transcripts did not start with AUG but rather with the codons CUG, GUG, ACG, or AUC. Truncated transcripts were also detected for U_L_8.5, U_L_12.5, U_L_24.5, U_L_26.5, U_S_1.5, and U_S_3.5. In the case of BoHV-1.1, similar transcription artifacts were also found throughout the entire genome [29,30,31]. For example, there are hundreds of potential alternative transcript start sites for both bICP22 and U_L_26.

Translating the ORF M sequence from the probable start codon to the end of the transcript elucidated in this study would produce a polypeptide of around 30 kDa. This would require the readthrough of several stop codons that exist in-frame after the highlighted stop codon in Figure 4. If, however, the first stop codon in the reading frame was functional, the estimated size would be closer to 13 kDa. In contrast, the homologous HSV-1 and HSV-2 proteins are translated past this position (where the canonical stop codon is observed in ORF M). The HSV-1 U_L_5.6 sequence has a single nucleotide difference so that the stop codon seen in ORF M is not a stop but a tyrosine codon instead (UAG to UAC). This transversion from cytosine to guanine residue could be part of the discrepancy between the transcript length and the protein product of ORF M. One plausible explanation would be that the stop codon is being misread by suppressor tRNAs. Suppressor tRNAs incorporate tyrosine, glutamine, leucine, cysteine, lysine, arginine, or tryptophan in the place of a stop codon depending on the organism and type of stop codon, as has been observed in viruses, prokaryotes, and eukaryotes [59,60,61,62]. The relative abundance of certain tRNAs, particularly tyrosine and tryptophan, has also been shown to influence translation efficiency in a tissue-specific manner, and increases in these tRNAs correlate with higher levels of stop codon readthrough (or SC-RT) [63]. Due to the size of the mature protein produced from ORF M and the improbability of a transversion mutation, we hypothesize that a readthrough event could be taking place. Supporting this hypothesis is the detection of what appear to be U_L_6 transcripts with long extended 5′ regions that include ORF M (Figure 2a; Tombácz et al., 2021 [31]). As shown in the dynamic transcriptome analysis by Tombácz and colleagues, these ORF M—U_L_6 extended transcripts increase in abundance over time, starting around 6 HPI (Figure 2b; Tombácz et al., 2021 [31]). This temporal pattern is in accordance with the detection of the ORF M protein via Western blot at about 8 h onward.

Recent research has explored the peptides produced by herpesvirus antisense transcripts [64,65]. While other studies have hypothesized that antisense transcripts could be a form of regulation of the sense transcript through siRNA [66], this seems to be more likely when the transcript is noncoding [66]. In this case, the ORF M transcript encodes a small protein, the function of which has yet to be determined (but is the focus of future studies). Xu and Ganem [65] have noted peptide-coding antisense transcripts in Kaposi Sarcoma Herpesvirus (KSHV), further suggesting that herpesviruses may have more regulatory mechanisms brought about by previously unknown peptides. Jaber and Yuan [64] have also revealed a small KSHV peptide (vSP-1) coded by the sequence antisense to ORF 50 (codes for replication and transcription activator—RTA) that interacts with RTA and protects it from degradation. Therefore, the vSP-1 peptide regulates KSHV gene expression by acting on its antisense protein, RTA. Yet another role for short ORF-encoded peptides may be immune related. Short ORF-encoded peptides can be efficiently incorporated and presented by MHC-I molecules, despite their low abundance and stability [67]. Our study shows that BoHV-1 also partakes in the production and translation of at least one antisense transcript. Further studies are needed to determine the function of ORF M during viral infection.

## Figures and Tables

**Table 1:** Table of primers used for qPCR, strand-specific RT-PCR, RACE, and 3′ primer walking.

Target	Forward Primer	Reverse Primer
bGH	5′ GCTTTCGCCCTGCTCTGCC 3′	5′ TCCTGCCTCCCCACCCCTA 3′
bICP0	5′ CGTTTGTGCGCAGCCTGTTG 3′	5′ GACGACGACTCTTCTGACTC 3′
RR	5′ TTTTACGAGACCGAGTGCCC 3′	5′ GACGAAAAGGTTGTGGGTGC 3′
gC	5′ TGATCGCAGCTATTTTCGCC 3′	5′ TTCTGGGCTACGAACAGCAG 3′
gB	5′ CTAACATGGAGCGCCGCTT 3′	5′ CGGGGCGATGCCGTC 3′
Histone H2A	5′ GTCGTGGCAAGCAAGGAG 3′	5′ GATCTCGGCCGTTAGGTACTC 3′
ORF M RT-PCR	5′ ATGAAGGCGTCTGGCACGAC 3′	5′ TTACTGCTGGTGGCTCATCAATGC 3′
ORF M qPCR	5′ GTTCGCGGGGTTGTTAATCAC 3′	5′ CCTCACCTGCCTTATCAC 3′
RACE	RACE Primer Provided by Invitrogen Nested Primer Provided by Invitrogen	1 5′ CCATGCCCGTCGTGGTCTGCGTGGGTT 3′ 2 5′ CCCACGCAGACCGACTCTCTCGAGTCCA 3′
Primer Walking	3 5′ ATGAAGGCGTCTGGCACGAC 3′	4 5′ CGGGCAGTTCGAGGCGCTGC 3′
		5 5′ CCAGCCCGCCCAGCATCCAG 3′ 6 5′ CCTACCACAGCCGTCACATCAACA 3′ 7 5′ CAAAAGCACCTGCATCCAAACCCTC 3′ 8 5′ CGCCGCTTGGCTGGTTCCGC 3′ 9 5′ AGCCGAGGTCTTCCTGAATTTCACT 3′

## Data Availability

Publicly available datasets were analyzed in this study. This data can be found here: https://www.ebi.ac.uk/ena Accession: PRJEB33511.

## Supplementary Materials

The following supporting information can be downloaded at: https://www.mdpi.com/article/10.3390/v15101977/s1, Figure S1: Derivative melt curve plot for ORF M primers. The derivative fluorescence detected from 60 to 100C is illustrated for mock-infected and cells infected for 2, 4, 6, and 8 hours. All infected samples display sharp peaks, and no distinct peak is produced by mock samples.; Figure S2: Multiple sequence alignment using full ORF M nucleotide sequence (divided in three panels).; Figure S3: Multiple sequence alignment using the full ORF M amino acid sequence.; Figure S4: Images of MDBK cells infected at various MOIs for 20 hours displayed at 100x, 400x, and 1000x (total) magnification. Images were captured with DAPI (blue), Texas Red (red) or FITC (green) filters. The phase contrast image to the right shows the appearance of cultured cells. Viral glycoprotein E was detected using an AlexaFluor594 secondary antibody (red), while ORF M was detected using an AlexaFluor488 conjugate secondary antibody (green). Nuclei were stained with DAPI stain (blue).; Figure S5: Amino Acid Conservation of UL5 Homologs and Antisense Sequences. Using the nucleotide sequence of the p-loop (phosphate binding region) of the helicase subunit coded by UL5 that overlaps ORF M and its homologs, this diagram illustrates how conservation of UL5 amino acid sequence does not necessarily transfer to the same conservation in the antisense amino acid sequence. Direction of each amino acid sequence is indicated with the gray arrow- shaped outline.; Figure S6: Visual representation of intergenic peptides detected by MS/MS that mapped to genomic coordinates 90,000-100,000 Peptides mapping to both direct and complement strands are shown. ORF M is differentiated from other complement peptides by the green diamond.
