# Supplementary material for: Evidence of a Protein-Coding Gene Antisense to the UL5 Gene in Bovine Herpesvirus I
Source: Viruses. 2023 Sep 22;15(10):1977. doi: 10.3390/v15101977 (PMC10610667; doi:10.3390/v15101977)
Supplement: Supplementary file 1 [file viruses-15-01977-s001.zip › viruses-2516306-supplementary.pdf]

Evidence of a protein-coding gene antisense to the U_L5 gene in Bovine Herpesvirus I

Victoria A. Jefferson[1], Hannah Bostick[1], Darby Oldenburg[2] and Florencia Meyer[1,*]

[1] Department of Biochemistry, Molecular Biology, Entomology & Plant Pathology, Mississippi State University, 32 Creelman St., Starkville, MS 39762, USA; vaj13@msstate.edu (V.A.J.); hannah.bostick.04@gmail.com (H.B.)
[2] Gundersen Medical Foundation, 1900 South Ave., La Crosse, WI 54601, USA; dgoldenb@gundersenhealth.org (D.O)
* Correspondence: florencia.meyer@msstate.edu

Supplementary Figures S1-S6

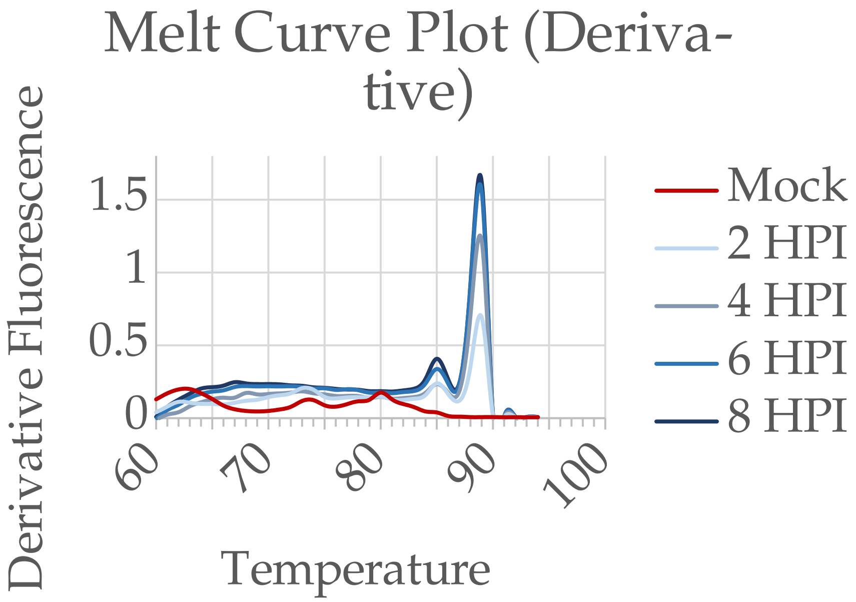

Supplementary Figure S1. Derivative melt curve plot for ORF M primers. The derivative fluorescence detected from 60 to 100C is illustrated for mock-infected and cells infected for 2, 4, 6, and 8 hours. All infected samples display sharp peaks, and no distinct peak is produced by mock samples.

S2.1

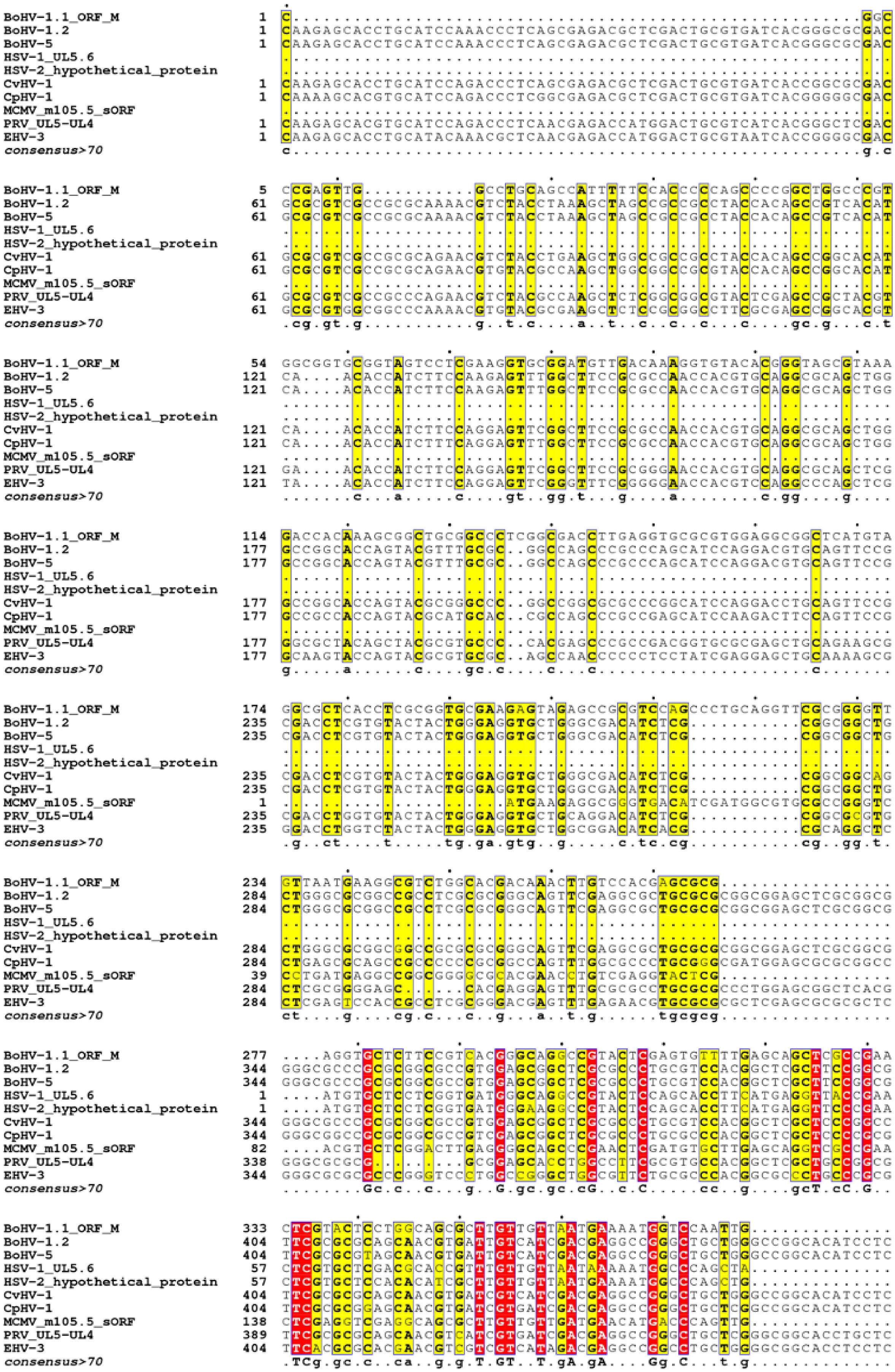
BoHV-1.1_ORF_M
BoHV-1.2
BoHV-5
HSV-1_UL5.6
HSV-2_hypothetical_protein
CvHV-1
CpHV-1
MCMV_m105.5_sORF
PRV_UL5-UL4
EHV-3
consensus>70

S2.2

```
BoHV-1.1_ORF_M              379 .......................................TGCGGGATCGCGGCGTACTCG
BoHV-1.2                    464 ACCGCGGTCGTTTACTGCTGGTGGCTCATCAATGCGGCGCACGATTCGGCGCAGTACCGC
BoHV-5                      464 ACCGCGGTCGTTTACTGCTGGTGGCTCATCAATGCGGCGCACGATTCGGCGCAGTACCGC
HSV-1_UL5.6                 103 .......................................TACGAGAGGCGGGCGTACTCG
HSV-2_hypothetical_protein  103 .......................................TGCGAGAGGCGCGTGTACTCG
CvHV-1                      464 ACCGCGGTCGTCTACTGCTGGTGGCTCATCAACGCGGCACACGACTCGGCGCAGTACCGC
CpHV-1                      464 ACCGCCGTCGTGTACTGCTGGTGGCTCATTAACGCGGCGTACGACTCCGCGCAGTACCGC
MCMV_m105.5_sORF            184 .......................................TGGGCGACGTCGCAGTACTCG
PRV_UL5-UL4                 449 ACGGCCGTCGTCTACTGCTGGTGGCTGATCAACGCCGCGTACGACACGACGCAGTACGCG
EHV-3                       464 ACGGTGGTGGTGTACTGCTGGTGGATGATCAACGCGGCCTACCGCTCCCCGCAGTACGTT
consensus>70                    .......................................tacga..cg.cgcaGTAC...

BoHV-1.1_ORF_M              400 CGCAGCGTGGGGTTGGTGATAAGGCAGGTGAGGACATTCTCGCTGGAGCGCACGCGGCAC
BoHV-1.2                    524 GACGGGGCCGTGCCCGTCGTGGTCTGCGTGGGTTCGCCCACGCAGACCGACTCTCTCGAG
BoHV-5                      524 GACGGGGCCATGCCCGTCGTGGTCTGCGTGGGTTCGCCCACGCAGACCGACTCTCTCGAG
HSV-1_UL5.6                 124 CGCAGCGTGCGGTTGCAGATGAGGTACGTGAGCACGTTCTCGCTCTGGCGGACGGAACAC
HSV-2_hypothetical_protein  124 CGTAGGGTGCGGTTGCAGATGAGGTACGTGAGCACGTTTTCGCTCTGCCGGACGGAGCAT
CvHV-1                      524 GACGGGCTCCGGCCCGTCGTGGTCTGCGTGGGCTCGCCCACGCAGACGGACTCGCTCGAG
CpHV-1                      524 GCGGGGGCGGCGCCGGTCGTCGTCTGCGTCGGCTCGCCGACGCAGACCGACTCGCTCGAG
MCMV_m105.5_sORF            205 GAGAGGACCGGGTCGCTGATGAGCGCGGAGAGGACGTCCATGCCGCGCTGCACGTCCCGA
PRV_UL5-UL4                 509 GCGCGCGCGCGCCCCGTGCTCGTCTGCGTGGGCTCGCCCACGCAGACGGACTCGCTCGAG
EHV-3                       524 GAGGGGCGCGTCCCCGTCATCGTCTGCGTGGGCTCTCCCACGCAGACGGACTCGCTCGAG
consensus>70                    g...Ggg....g.c.gt..T...ct.cGtg.G..Cg.ccacGC.g.....c.Cgc.c.a.

BoHV-1.1_ORF_M              460 TTTAGCTTGTGGTGCTCGAAGGTGGACTCGAGAGAGTCGGTCTGCGTGGGCGAACCCACG
BoHV-1.2                    584 TCCACCTTCGAGCACCACAAGCTAAAGTGCCGCGTGCGCTCCAGCGAGAATGTCCTCACC
BoHV-5                      584 TCCACCTTCGAGCACCACAAGCTAAAGTGCCGCGTGCGCTCCAGCGAGAATGTCCTCACC
HSV-1_UL5.6                 184 CGCAGTTTCTGGTGCTCGAAGGTCGACTCCAGGGACGCCGTCTGCGTCGGCGAGCCCACA
HSV-2_hypothetical_protein  184 CGCAGTTTTTGGTGTTCGAAGGTGGACTCCAGCGAGGCCGTCTGGGTCGGCGACCCCACG
CvHV-1                      584 TCCACCTTCGAGCACCAGAACCTGAAGTGCCGCGTGCGCTCCAGCGAGAACGTCCTCACC
CpHV-1                      584 TCCACATTTGAGCACCAGAACCAAAAGTGCCGCGTGCGCTCCAGCGAGAACGTCCTAACG
MCMV_m105.5_sORF            265 TTCTGCGTGCGGTGGTCGTAG.......................................
PRV_UL5-UL4                 569 TCGCGCTTCGAGCACGCGCGGCAGCTGTGCCGCGTGCGCGCCAGCGAGAACCTGCTCACG
EHV-3                       584 TCCCGCTTTGAGCACAAAAACCTCAAGTGCCACGTGCGGTCGAGCGAAAACGTCCTCACG
consensus>70                    t.ca.ctT...G..c..gaag.t..a.t.c.gcg.g..c..c.gcg....cg..c.cac.

BoHV-1.1_ORF_M              520 CAGACCACGACGGGCATGGCCCCGTCGCGGTACTGCGCCGAATCGTGCGCCGCATTGATG
BoHV-1.2                    644 TGCCTCATCACCAACCCCACGCTGCGCGAGTACGCCGCGATCCCGCACAATTGGACCATT
BoHV-5                      644 TGCCTTATCACCAACCCCACGCTGCGCGAGTACGCCGCGATCCCGCACAATTGGACCATT
HSV-1_UL5.6                 244 CACACCAACACGGGCCGCAGGCGGGCCGCGTACTGGGGGGTGTGGTACAGGGCGTTAATC
HSV-2_hypothetical_protein  244 CACACCAGCACCGGCCGCAGGCGGCCCGCGTACTGGGGGGTGTGGTACAGGGCGTTAATC
CvHV-1                      644 TGCCTCATCACCAACCCCACGCTGCGCGAGTACGCCGCGGTCCCGCGCAACTGGACCATC
CpHV-1                      644 TGCCTGATTACCAACCCGACCCTCCGCGAGTACGCCTCCGTCCCGCACAACTGGACCATC
MCMV_m105.5_sORF                ............................................................
PRV_UL5-UL4                 629 TACCTGATCACCAACCGCGCGCTGCGCGAGTACACGGACCTCTCGCGCAACTGGGCCATC
EHV-3                       644 CACATCATCACCAGCCGGACCCTCCGGGAGTACGCGTCGCTCTCCCGGAACTGGGCCATC
consensus>70                    ..c...a.cacc..cc..ac.c.gc.cg.gtac...g...t..cg..ca....g...at.

BoHV-1.1_ORF_M              580 AGCCACCAGCAGTAAACGACCGCGGTGAGGATGTGCCGGCCCAGCAGCCCGGCCTCGTCG
BoHV-1.2                    704 .TTCATTAACAACAAGC.GCTGCCAGGAGTACGAGTTCGGCGAGCTGCTCAAAACGCTCG
BoHV-5                      704 .TTCATTAACAACAAGC.GCTGCCAGGAGTACGAGTTCGGCGAGCTGCTCAAAACACTCG
HSV-1_UL5.6                 304 ATCCACCAGCAATACACCACGGCCGTGAGGAGGTGA........................
HSV-2_hypothetical_protein  304 ATCCACCAGCAATACACCACGGTCGTGAGTAGGTGCCGCCCCAGGAGCCCGGCCTCGTCG
CvHV-1                      704 .TTCATCAACAACAAGC.GCTGCCAGGAGTACGAGTTCGGCGAGCTCCTCAAGACGCTCG
CpHV-1                      704 .TTTATCAACAACAAGC.GCTGCCAGGAGTACGAGTTCGGGGAGCTCCTCAAGGTGCTCG
MCMV_m105.5_sORF                ............................................................
PRV_UL5-UL4                 689 .TTCATCAACAACAAGC.GCTGCCAGGAGTACGAGTTCGGCGAGCTCATGAAGGCGCTCG
EHV-3                       704 .TTCATCAACAACAAGC.GCTGCCAGGAGTACGAGTTCGGGGAGCTCATGAAGGTGCTCG
consensus>70                    .t.ca.ca.caa.aa.c..c.gcc..gagta.g.g...g...agc............tcg

BoHV-1.1_ORF_M              640 ATGACAATCACGTTGCTACGCGCGAACGCCGGAAGTGAGCCGTGGACGCAGGGCGCGAGC
BoHV-1.2                    762 AGTACGGCCTGCCCGTGACGGAAGAGCACC..............................
BoHV-5                      762 AGTACGGCCTGCCCGTGACGGAAGAGCACC..............................
HSV-1_UL5.6                     ............................................................
HSV-2_hypothetical_protein  364 ATGACGATAATGTTGCTGCGGGTGAAAGCCGGCAGCGCCCCGTGTGTGACCGAGGCCAGG
CvHV-1                      762 AGTACGGCCTGCCCGTGACGGAGGAGCACC..............................
CpHV-1                      762 AGTACGGCTTGCCGGTAACGGAGGAGCACC..............................
MCMV_m105.5_sORF                ............................................................
PRV_UL5-UL4                 747 AGTACGGCCTCCCGCTGACGGACGAGCACC..............................
EHV-3                       762 AGTACGGACTGCCCATCACCGAGGAGCACA..............................
consensus>70                    a..acg...........acgg..ga.c.cc..............................

BoHV-1.1_ORF_M              700 CGCTCCACGGCGCCGCGCGGGCGCCCCGCCGCGAGCTCCGCCGCGCGCAGCGCCTCGAAC
BoHV-1.2                    792 ...................TCGCGCTCGTGGACAAGTTTGTCGTGCCAGACGCCTTCATT
BoHV-5                      792 ...................TCGCGCTCGTGGACAAGTTTGTCGTGCCAGACGCCTTCATT
HSV-1_UL5.6                     ............................................................
HSV-2_hypothetical_protein  424 CGCGTGAGGGCACCCTGGCCCAGCCCCAAAGTCTGCTCTAGGGCGGTGAGGGCGTGGAAC
CvHV-1                      792 ...................TCGCGCTCGTGGACAAGTTCGTCGTGCCCGACGCCTTCATC
CpHV-1                      792 ...................TCGCGCTGGTCGACAAGTTCGTCGTCCCCGACGCGTACATC
MCMV_m105.5_sORF                ............................................................
PRV_UL5-UL4                 777 ...................TGCGCCTCGTCGACAGCTTCGTGGTGCCCGAGGCCTACATC
EHV-3                       792 ...................TGCGCATGGTGGACAACTTTGTGGTCCCCGAGGCGTACATC
consensus>70                    ........................c..g..g.ca..t..g..g..c.....gc.t..a..

BoHV-1.1_ORF_M              760 TGCCCGCGCGAG.........GCGGCCGCGCCCAGCAGCCGCCGCGAGATGTCGCCCAGC
BoHV-1.2                    833 AACAACCCCGCG.....................AACCTGCAGGGCTGGACGCGGCTCTAC
BoHV-5                      833 AACAACCCCGCG.....................AACCTGCAGGGCTGGACGCGGCTCTAC
HSV-1_UL5.6                     ............................................................
HSV-2_hypothetical_protein  484 TCGTTTCGCGCGTCTTCGCCCCCGTGCGCCGCCAGGCCCCGCTTGGTGATGTCGAGGATC
CvHV-1                      833 AACAACCCCGCG.....................AACCTGCAGGGCTGGACGCGGCTCTAC
CpHV-1                      833 AACAACCCCGCG.....................AACCTGCAGGGCTGGACGCGGCTCTAC
MCMV_m105.5_sORF                ............................................................
PRV_UL5-UL4                 818 AACAACCCGGCG.....................AACCTGCAGGGCTGGACGCGCCTGTAC
EHV-3                       833 AACAACCCCGCC.....................AACCTACCCGGCTGGACGCGGCTGTAC
consensus>70                    ..c...c.cgcg.....................a.c...c...gc..ga.g..gc....c
```

S2.3

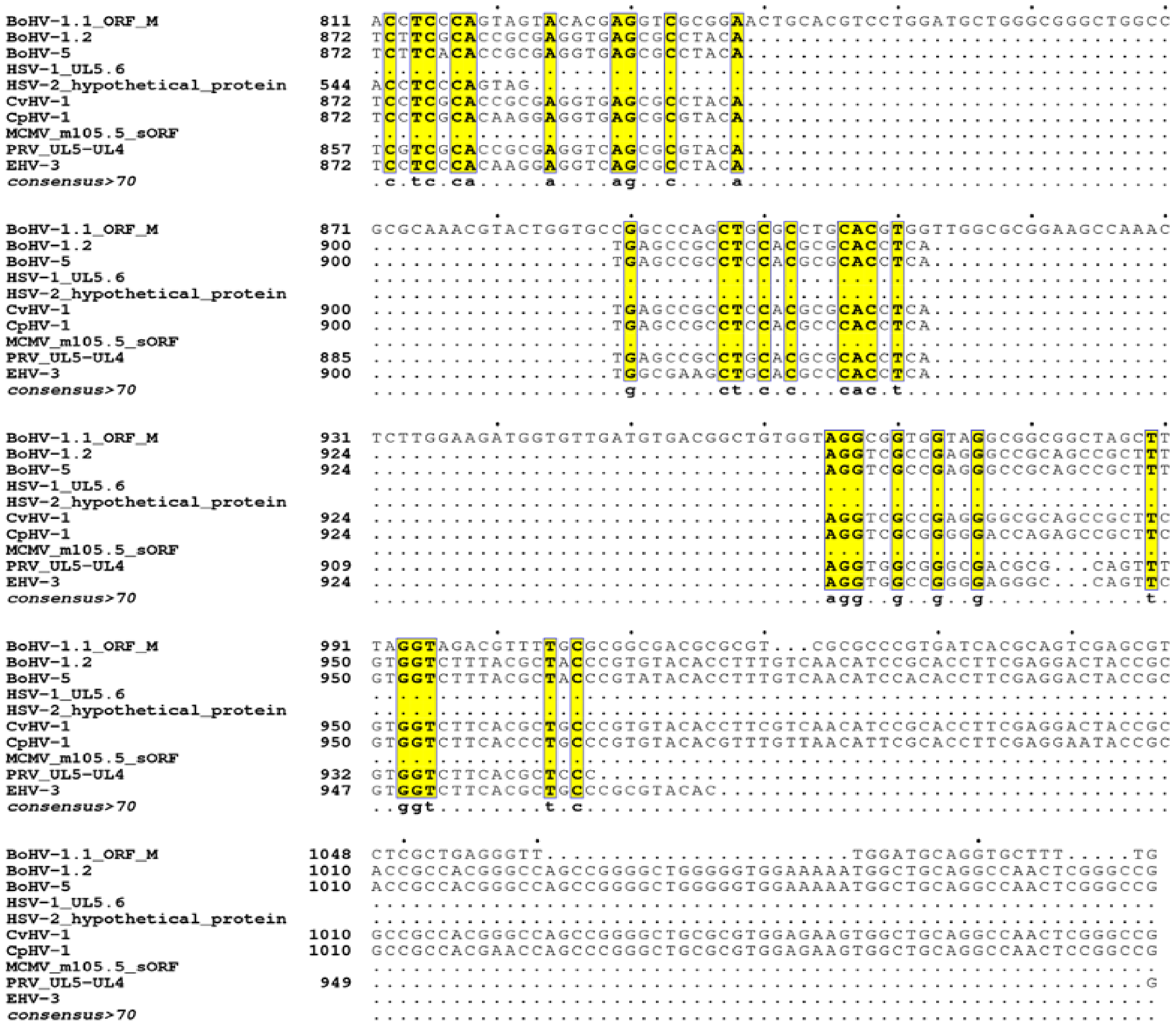

Supplementary Figure S2. Multiple sequence alignment using full ORF M nucleotide sequence (divided in three panels).

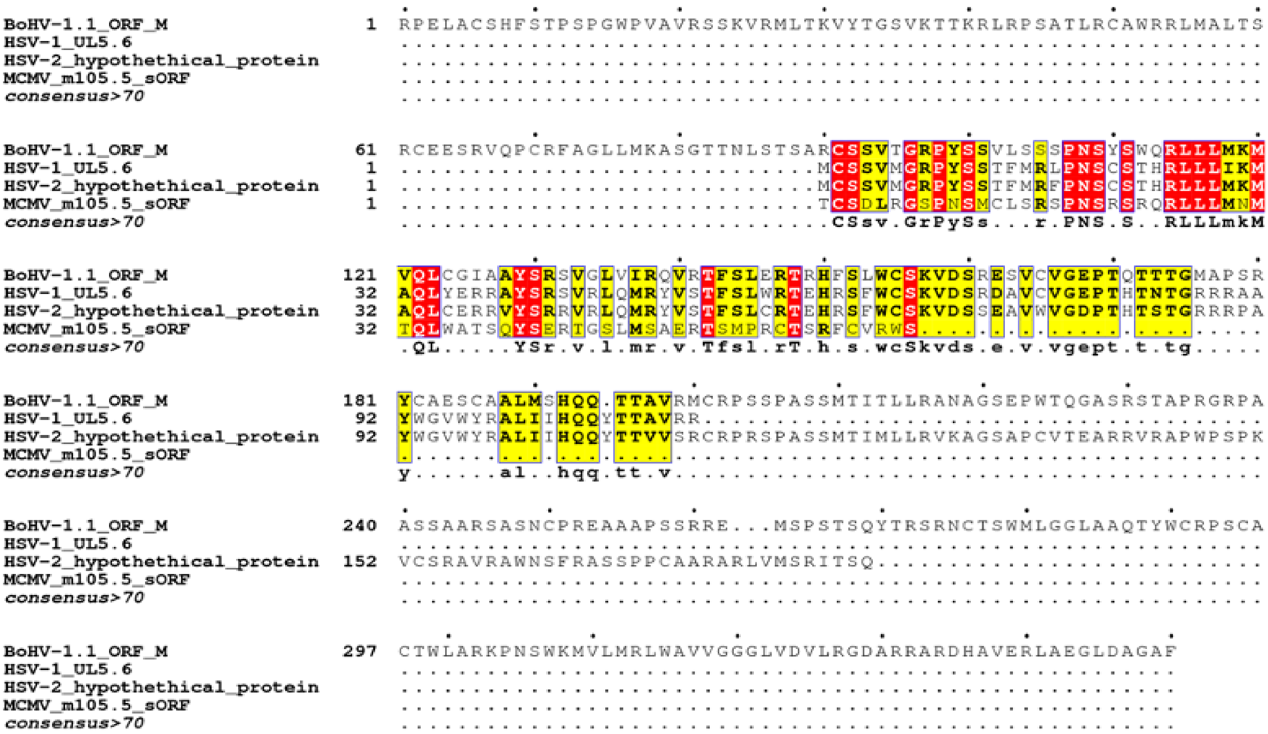

Supplementary Figure S3. Multiple sequence alignment using the full ORF M amino acid sequence.

S4.1

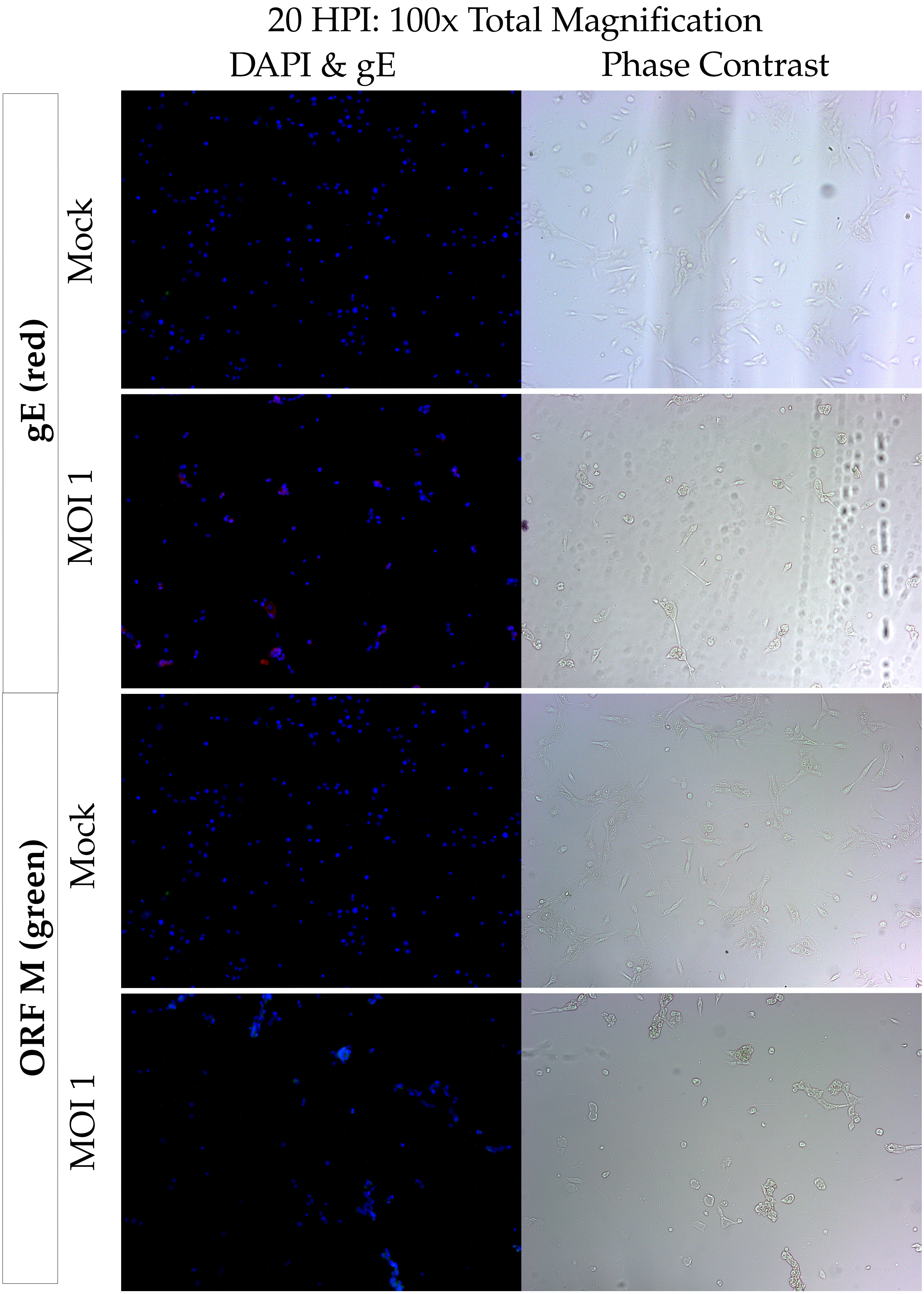
20 HPI: 100x Total Magnification
DAPI & gE
Phase Contrast
gE (red)
Mock
MOI 1
ORF M (green)
Mock
MOI 1

S4.2

20 HPI: 400x Total Magnification

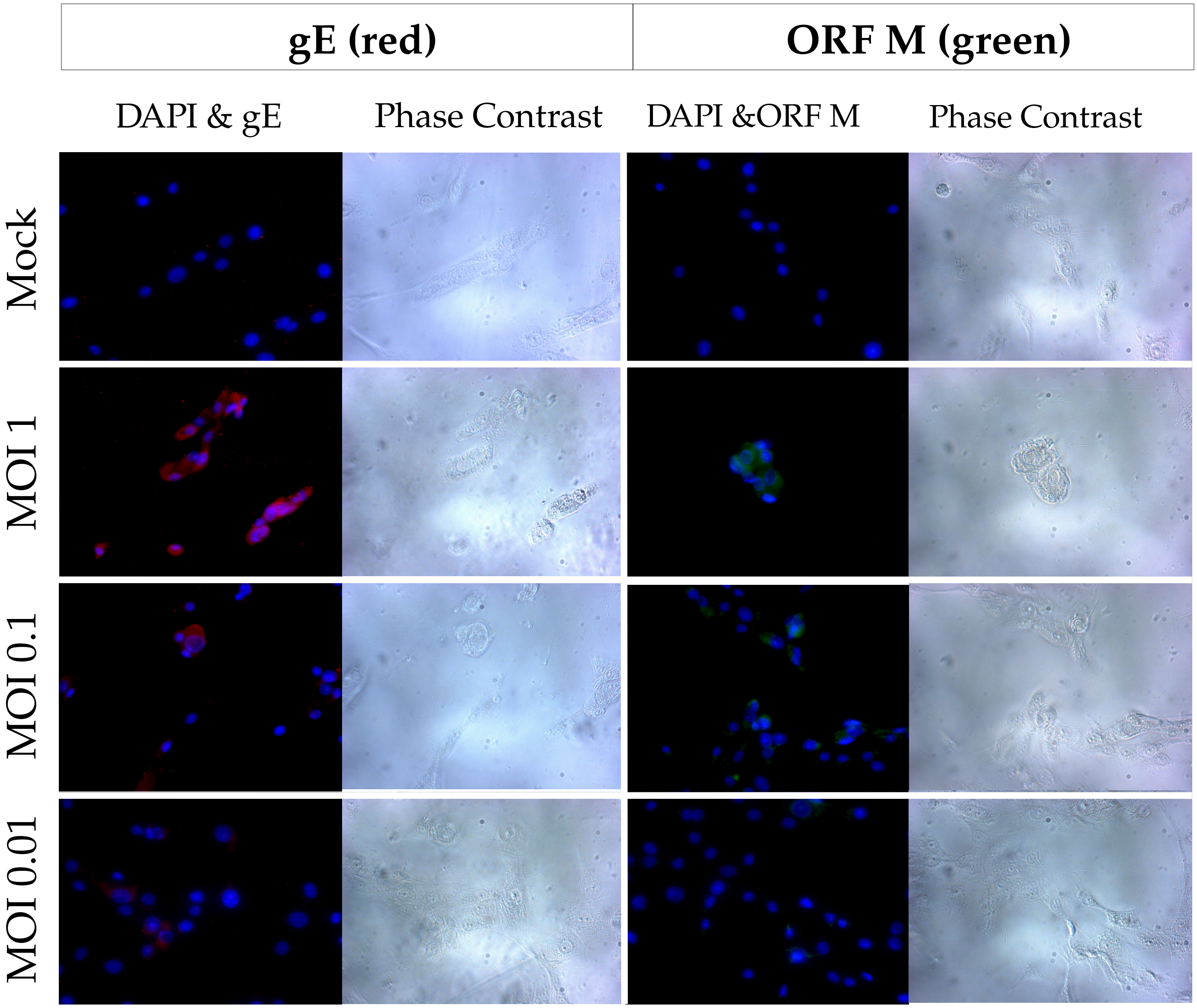

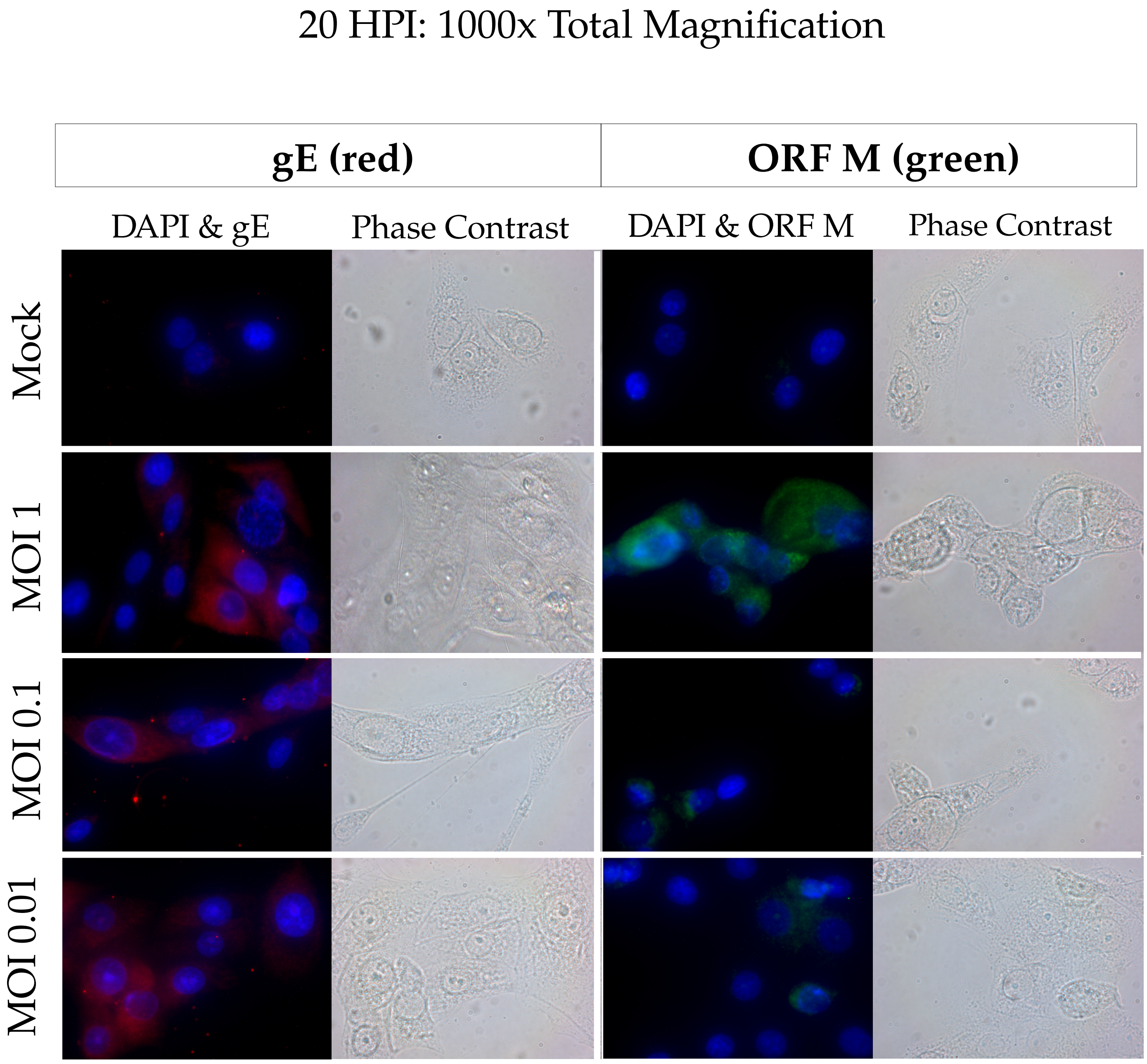

Supplementary Figure S4. Images of MDBK cells infected at various MOIs for 20 hours displayed at 100x, 400x, and 1000x (total) magnification. Images were captured with DAPI (blue), Texas Red (red) or FITC (green) filters. The phase contrast image to the right shows the appearance of cultured cells. Viral glycoprotein E was detected using an AlexaFluor594 secondary antibody (red), while ORF M was detected using an AlexaFluor488 conjugate secondary antibody (green). Nuclei were stained with DAPI stain (blue).

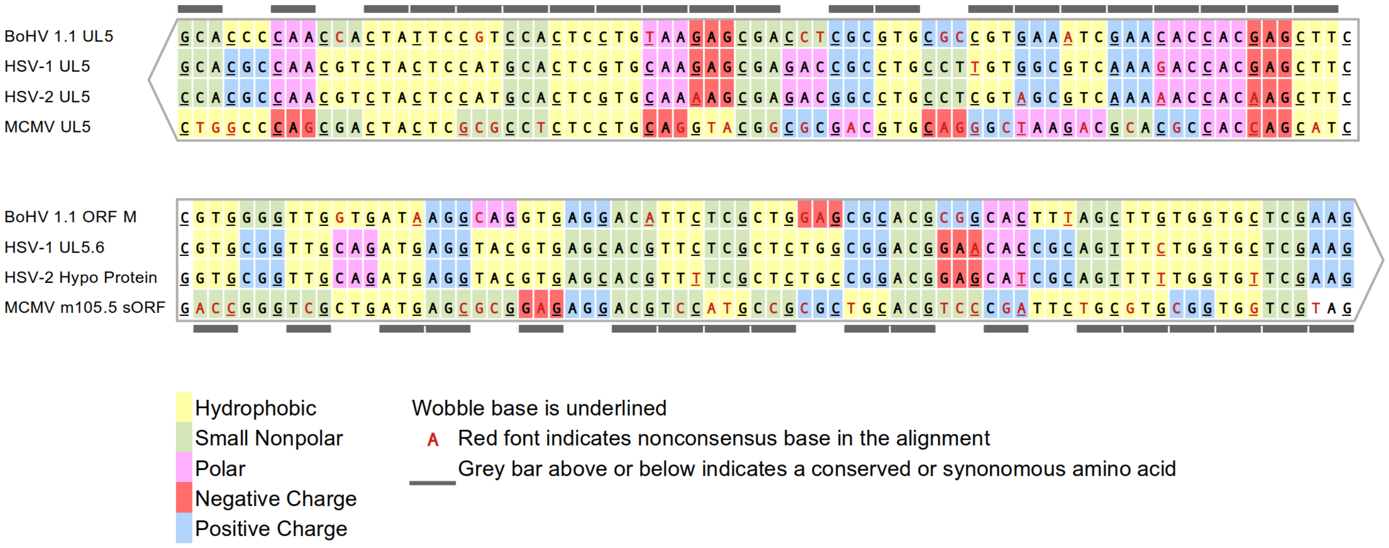

Supplementary Figure S5. Amino Acid Conservation of U_L5 Homologs and Antisense Sequences. Using the nucleotide sequence of the p-loop (phosphate binding region) of the helicase subunit coded by UL5 that overlaps ORF M and its homologs, this diagram illustrates how conservation of UL5 amino acid sequence does not necessarily transfer to the same conservation in the antisense amino acid sequence. Direction of each amino acid sequence is indicated with the gray arrow-shaped outline.

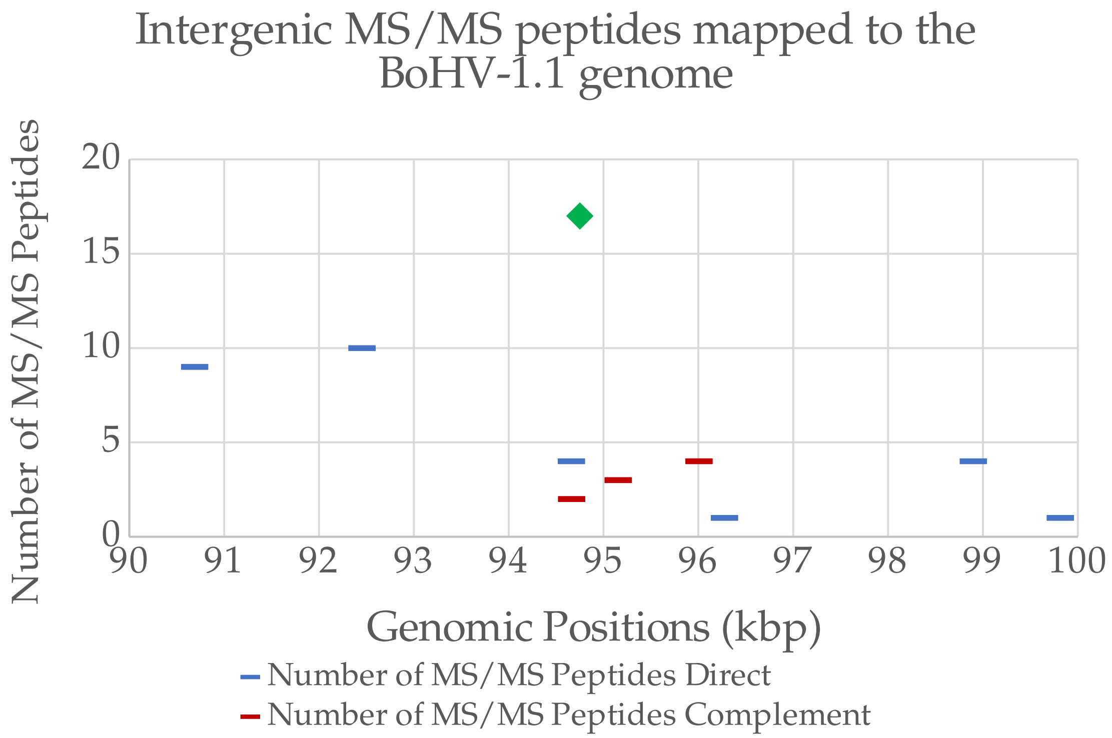

Supplementary Figure S6. Visual representation of intergenic peptides detected by MS/MS that mapped to genomic coordinates 90,000-100,000 Peptides mapping to both direct and complement strands are shown. ORF M is differentiated from other complement peptides by the green diamond.
